# Scientific News

**Published:** 1880-02

**Authors:** Edgar D. Swain


### Scientific News.

Herbert Spencer's "Data of Ethics" has recently been issued from the press, and the reception it has met from the critics, is so contrary to the usual course pursued, that could the knowledge of it be carried back to the good old times of Pope and Dr. Johnson, it would be placed on record as a literary eighth wonder. The *Popular Science Monthly* of December last, says that "its reception by critics generally, has been most gratifying, and indicates a favorable change in the habits of these parties. Formerly, they seem to have been anxious to put before the world, their own views of Spencer's works; now they conclude it is better to let him speak for himself." "Even Prof. Bain, whose position certainly entitles him to assume the functions of judge, is chiefly concerned to get Spencer fully and fairly before his readers." Mindful of this fact the *Monthly* of same date as above, reprints from *Mind*, Prof. Bain's able *resume*, which, by the way, well repays perusal. The publication of the "Ethics" was premature and not in the order intended, for the author says: "I have been thus led to deviate from the order originally set down by the fear that persistence in conforming to it might result in leaving the final work of the series unexecuted." Again, "to leave this purpose unfulfilled' after making so extensive a preparation for fulfilling it, would be a failure, the probability of which I do not like to contemplate." Prof. Bain says Spencer regards the work "as the end and out-come of all his labors; the object to whieh all the preceeding parts of his systematic elaboration are preparatory."

---

The most powerful telescope in existence is the magnificent new reflecting telescope which has just been finished by Mr. A. Ainslie Common, and is erected at his residence, at Ealing, (England). This telescope has a silver-on-glass speculum, thirty-seven and a half inches in diameter, and a focal length of just over twenty feet. It is equitorially mounted in a novel but efficacious manner, and is driven by a powerful clock, controlled in an ingenious manner by a method invented by Mr. Common. This new telescope, which has only been finished a month, has turned out a great success, and is unquestionably the finest and most powerful telescope in existence. E. Neison, in the Popular Science Monthly for January.

---

HERR. ADLER, a German, has perfected a multiplying process, in which a plate or film of gelatine is etched by a concentrated solution of alum. The paper upon which the "alum writing" or drawing is made, is placed over the gelatine, and when the latter has been acted upon, the copy can be reproduced many times with the assistance of common printing ink spread over the gelatine plate.

---

In an address delivered at St. George's Hospital, London, W. B. Dalby makes a strong argument for the inductive method of study. He insists upon the acquirement of knowledge directly from nature, so that a statement may be subjected to rigid tests to insure its veracity. The tendency of modern investigation is to scrupulous accuracy, as upon the truthfulness of details, depends the value of our whole system of scientific research.

---

Dr. Daniel Draper says the outfit for a working meteorological observatory need only consist of seven pieces of apparatus, viz: 1. Barometer. 2. Dry and wet metallic thermometers. 3. Sun thermometer. 4. Instrument for recording the direction of the wind. 5. Instrument for recording the velocity of the wind, 6. Instrument for recording the force of the wind. 7. Rain gauge.

---

The population of Africa is estimated at 172,250,000, disposed as follows: Negroes, 130,000,000; Hamites, 20,000,000; Bantus, 13,000,000; Foolahs, 8,000,000; Nubians, 1,500,000; Hottentots-50,000. These estimates, however, can not be anything but approximate, as it is manifestly impossible to make an accurate census.

---

M. Vioble gives the following as the exact degrees of heat at which five of the refactory metals fuse: Silver, 1.749° Fah. Gold, 1.863°. Copper, 1.890°. Platinum, 3.195°. Iridium, 3.510°.

